# Peripheral neuropathy via mutant tRNA synthetases: Inhibition of protein translation provides a possible explanation

**DOI:** 10.1002/bies.201600052

**Published:** 2016-06-28

**Authors:** Erik Storkebaum

**Affiliations:** ^1^ Molecular Neurogenetics LaboratoryMax Planck Institute for Molecular BiomedicineMünsterGermany; ^2^ Faculty of MedicineUniversity of MünsterMünsterGermany

**Keywords:** aminoacylation, animal model, axonal degeneration, Charcot‐Marie‐Tooth peripheral neuropathy, gain‐of‐toxic‐function, translation, tRNA synthetase

## Abstract

Recent evidence indicates that inhibition of protein translation may be a common pathogenic mechanism for peripheral neuropathy associated with mutant tRNA synthetases (aaRSs). aaRSs are enzymes that ligate amino acids to their cognate tRNA, thus catalyzing the first step of translation. Dominant mutations in five distinct aaRSs cause Charcot‐Marie‐Tooth (CMT) peripheral neuropathy, characterized by length‐dependent degeneration of peripheral motor and sensory axons. Surprisingly, loss of aminoacylation activity is not required for mutant aaRSs to cause CMT. Rather, at least for some mutations, a toxic‐gain‐of‐function mechanism underlies CMT‐aaRS. Interestingly, several mutations in two distinct aaRSs were recently shown to inhibit global protein translation in *Drosophila* models of CMT‐aaRS, by a mechanism independent of aminoacylation, suggesting inhibition of translation as a common pathogenic mechanism. Future research aimed at elucidating the molecular mechanisms underlying the translation defect induced by CMT‐mutant aaRSs should provide novel insight into the molecular pathogenesis of these incurable diseases.

**Abbreviations**
aaRS: aminoacyl tRNA synthetase
ALS: amyotrophic lateral sclerosis
CMT: Charcot‐Marie‐Tooth
CMT‐aaRS: CMT associated with mutations in tRNA synthetases
CMT2D: CMT type 2D
CMT2N: CMT type 2N
DI‐CMTC: dominant intermediate CMT type C
ENU: N‐ethyl‐N‐nitrosourea
GAIT: interferon‐gamma‐activated inhibitor of translation
HMN5A: distal hereditary motor neuropathy type Va
iPSC: induced pluripotent stem cell
MSC: multi‐synthetase complex
NCAT: non‐canonical amino acid tagging
NCV: nerve conduction velocity
NMJ: neuromuscular junction
Nrp1: neuropilin‐1
WT: wild type

## Introduction: Aminoacyl tRNA synthetases catalyze the first step of protein synthesis

**Figure 1:**
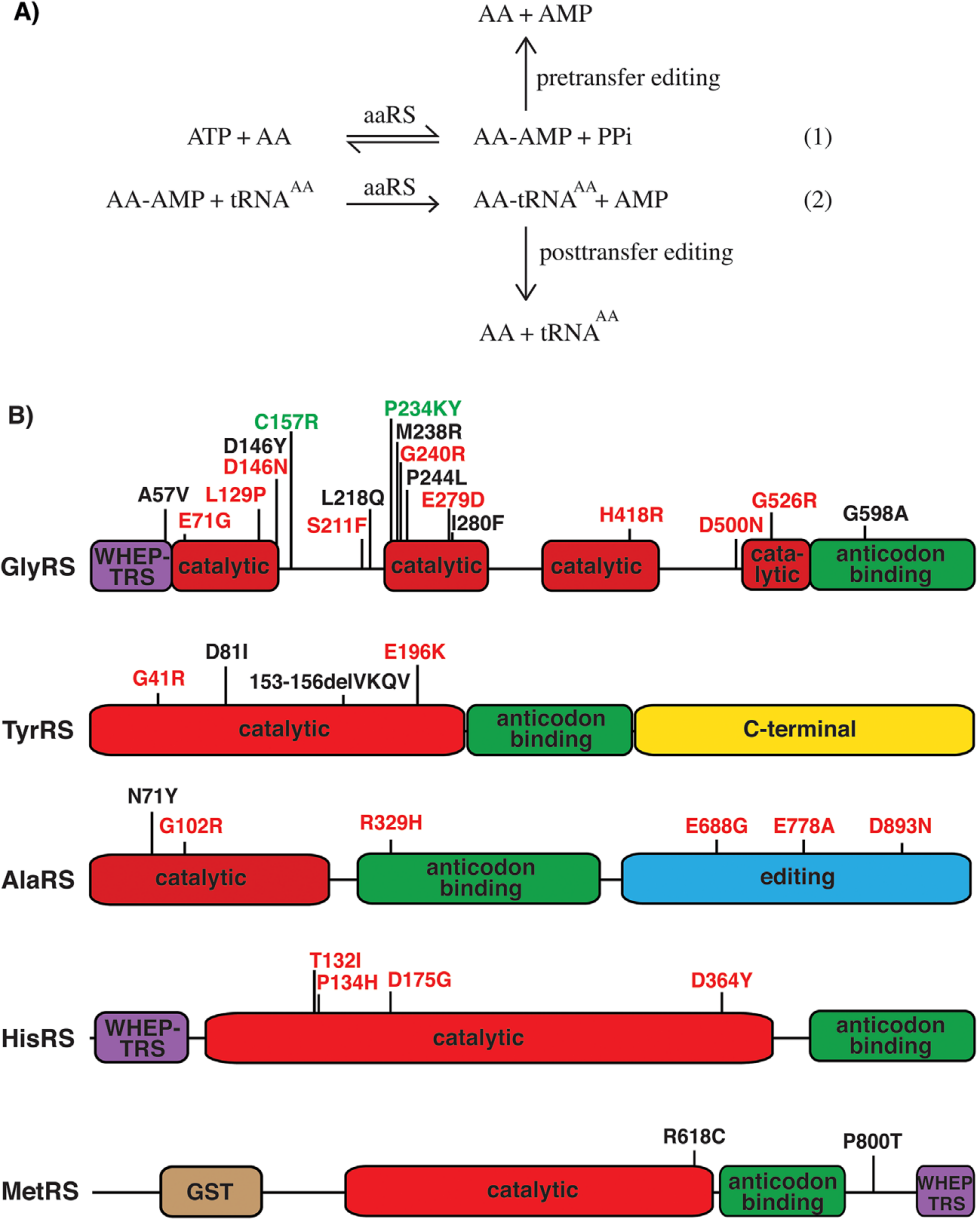
Mutations in tRNA synthetases cause CMT peripheral neuropathy. **A:** aaRSs catalyze tRNA aminoacylation in a two‐step reaction. In the first step (1), the amino acid (AA) is activated with ATP, resulting in the formation of an enzyme‐bound aminoacyl‐adenylate (AA‐AMP), with the concomitant release of pyrophosphate (PPi). At this stage, aaRSs with pre‐transfer editing activity can remove misacetylated aminoacyl adenylate. In the second step (2), the aminoacyl‐adenylate is transferred to the tRNA, with release of AMP. aaRSs which posses posttransfer editing activity can hydrolyze misacetylated tRNAs to prevent amino acid misincorporation in nascent proteins. **B:** Distribution of CMT‐associated mutations in aaRSs. Schematic representation of GlyRS, TyrRS, AlaRS, HisRS, and MetRS proteins and their functional domains. CMT‐associated mutations found in single patients are indicated in black, while mutations that co‐segregate with disease in CMT families are labeled red. Mutations labeled green are equivalent to mutations in mouse CMT2D models. For GlyRS, the positions of the mutations refer to the cytoplasmic form of the human protein.

Protein translation involves the matching of triplet codons in the mRNA with anticodons of tRNAs. This job is performed by the ribosome, which subsequently transfers the nascent peptide chain to the amino acid attached to a matching tRNA. Therefore, the accuracy of the amino acid sequence of a protein depends on three factors: the flawlessness of the mRNA coding sequence, the correct matching of codon and anticodon by the ribosome, and the correct attachment of amino acids to the tRNA. Aminoacyl tRNA synthetases (aaRSs) are the enzymes that catalyze the covalent attachment of amino acids to their cognate tRNAs in a two‐step reaction (Fig. 1A) 1, 2. After synthesis, aminoacyl‐tRNAs are delivered to the ribosome by elongation factors. It is thought that during the translation cycle, tRNAs are always chaperoned by aaRSs, elongation factors, or other proteins that directly interact with aaRSs, and never freely diffuse in the cytoplasm of mammalian cells 3.

Cytoplasmic aaRSs constitute a family of 19 enzymes, one for each amino acid, with the exception of the bifunctional glutaminyl‐prolyl‐tRNA synthetase. Mitochondrial aaRSs are usually encoded by separate genes, except for GlyRS and LysRS, for which the cytoplasmic and the mitochondrial enzymes are encoded by a single gene and generated by the use of alternative translational start sites or by alternative mRNA splicing 4, 5, 6. aaRSs are grouped into two structural classes (I and II) based on the architecture of the catalytic core domains (Table 1) 7, 8. Class I aaRSs are monomeric or homodimeric, and TyrRS and TrpRS function as obligate homodimers, in which the tRNA binds across the subunits 9 (Table 1). Most class II aaRSs are homodimeric. Each aaRS class is further subdivided into subclasses (Table 1) 10, 11.

**Table 1:** Classification and properties of human cytoplasmic tRNA synthetases

aaRS	Class	Editing activity	Editing domain	Quaternary structure^a^	MSC member
IleRS	I a	Yes	Yes	MSC	Yes
ValRS	I a	Yes	Yes	α	No
LeuRS	I a	Yes	Yes	MSC	Yes
**MetRS**	**I a**	**Yes**	**No** ^b^	**MSC**	**Yes**
CysRS	I a	No	No	α	No
ArgRS	I a	No	No	MSC	Yes
GluRS^c^	I b	No	No	MSC	Yes
GlnRS	I b	No	No	MSC	Yes
**TyrRS**	**I c**	**No**	**No**	**α_2_**	**No**
TrpRS	I c	No	No	α_2_	No
ProRS^c^	II a	Yes	Yes	MSC	Yes
ThrRS	II a	Yes	Yes	α_2_	No
**GlyRS**	**II a**	**No**	**No**	**α_2_**	**No**
SerRS	II a	Yes	No^b^	α_2_	No
**HisRS**	**II a**	**No**	**No**	**α_2_**	**No**
LysRS	II b	Yes	No^b^	MSC	Yes
AspRS	II b	No	No	MSC	Yes
AsnRS	II b	No	No	α_2_	No
**AlaRS**	**II c**	**Yes**	**Yes**	**α**	**No**
PheRS	II c	Yes	Yes	α_2_β_2_	No

Enzymes marked in bold have been implicated in CMT.

^a^ α: monomer; α_2_: dimer; α_2_β_2_: heterotetramer; MSC: member of the multi‐synthetase complex.

^b^ These enzymes only possess pre‐editing activity.

^c^ In humans, GluRS and ProRS activities are contained within a single bifunctional protein, designated GluProRS.

All aaRSs contain a catalytic and an anticodon binding domain, which contacts the anticodon of the cognate tRNA. A number of aaRSs further contain dimerization or multimerization domains, editing domains, which mediate editing of mischarged tRNAs (Box 1), protein interaction domains, and domains responsible for subcellular localization. Apart from their canonical tRNA aminoacylation function, several aaRSs acquired additional functional domains during evolution, which mediate non‐canonical functions in a diversity of biological processes, including inflammation, transcriptional regulation, translational regulation, apoptosis, rRNA transcription, angiogenesis, cell‐signaling, autoimmune response, tRNA maturation, and mitochondrial RNA splicing 10, 12, 13. Finally, nine cytoplasmic aaRSs and three auxiliary proteins (aaRS‐interacting multi‐functional proteins or AIMPs) associate to form the “multi‐synthetase complex” (MSC) (Table 1), which may facilitate protein translation. In addition, some MSC components are released from the MSC upon specific signals, whereupon they exert non‐canonical activities 10, 14.

Box 1 **tRNA editing by aaRSs**

The accuracy of tRNA aminoacylation is essential for correct translation of mRNA triplet codons into the primary amino acid sequence of proteins. This requires correct discrimination of both the amino acid and the tRNA by the aaRS. Apart from recognition of the tRNA anticodon by the anticodon binding domain of the aaRS, several additional interactions between aaRS and tRNA typically occur, enabling correct recognition of the cognate tRNA 2, 10. It can be much more challenging for aaRSs to discriminate between cognate and structurally similar, non‐cognate amino acids 10. Therefore, 10 of 19 cytoplasmic aaRSs also possess proofreading or editing activity (Table 1). Two modes of editing can be distinguished: (i) pre‐transfer editing, which removes misactivated aminoacyl adenylate that is produced in the first step of the aminoacylation reaction; and (ii) post‐transfer editing, which involves hydrolysis of mis‐aminoacylated tRNAs (Fig. 1A) 10, 11, 98. Post‐transfer editing requires the presence of a spatially separate editing domain, which is present in 7 of 10 editing aaRSs. SerRS, MetRS, and LysRS lack such a domain and catalyze pre‐transfer editing only (Table 1). The discovery of editing activity by aaRSs has led to the “double‐sieve” model to explain the accuracy of tRNA aminoacylation: the active site serves as the first sieve, activating cognate, isosteric, and smaller amino acids but excluding larger amino acids, and the editing site is the second sieve, hydrolyzing misactivated or mischarged amino acids but rejecting cognate amino acids 10, 98.

## Dominant mutations in tRNA synthetase genes cause Charcot‐Marie‐Tooth peripheral neuropathy

Over the past 12 years, heterozygous mutations in five distinct tRNA synthetase genes have been identified as a genetic cause of Charcot‐Marie‐Tooth (CMT) peripheral neuropathy. This genetic disorder is characterized by selective degeneration of peripheral motor and sensory axons, leading to progressive muscle weakness and wasting, and decreased sensation of vibration, touch, and pain. CMT patients typically display reduced or absent deep‐tendon reflexes and foot deformities such as high arches (pes cavus) and hammer toes. Disease onset is variable, but usually occurs in the first two decades of life and subsequently shows a slow progression over decades, patients ultimately becoming wheelchair‐bound. Typically, distal muscles are affected first, with a disto‐proximal progression over time 15. With a prevalence of one in 2,500 individuals, CMT is the most common inherited neuromuscular disorder 16.

Traditionally, a distinction is made between demyelinating forms of CMT (CMT1) and axonal forms (CMT2). Clinically, this distinction is based on the measurement of median or ulnar nerve conduction velocities (NCVs): severely reduced NCVs of <38 m/s are classified as CMT1, whereas normal or mildly reduced NCVs (>38 m/s) are classified as CMT2 17. CMT1 is pathologically characterized by segmental demyelination and remyelination with formation of so‐called onion bulbs: concentric arrangements of supernumerary Schwann cells around an incompletely remyelinated axon 18. The primary demyelination in CMT1 ultimately leads to axonal degeneration, giving rise to the classical CMT symptoms 19. CMT1 is the most prevalent, while CMT2 accounts for ∼20% of cases 20. CMT2 is electrophysiologically characterized by reduced compound action potential amplitudes, and pathologically by chronic axonal degeneration and regeneration 19. More recently, the existence of intermediate forms of CMT has been recognized, characterized by intermediate NCVs (25–45 m/s), and pathological features of both demyelination and axonal degeneration 21. CMT is not only clinically, but also genetically heterogeneous: mutations in more than 70 genes have been identified as causative for CMT 22.

aaRSs were first implicated in CMT in 2003, when four heterozygous mutations in *GARS* (E71G, L129P, G240R, G526R), encoding cytoplasmic GlyRS, were reported as causative for CMT type 2D (CMT2D) and distal hereditary motor neuropathy type Va (HMN5A) 23. HMN5A is phenotypically similar to axonal CMT, but without sensory involvement 24. A series of follow‐up studies reported additional mutations (A57V, D146N, D146Y, S211F, L218Q, M238R, P244L, E279D, I280F, H418R, D500N, G598A), adding up to 16 distinct mutations 25, 26, 27, 28, 29, 30, 31, 32, 33, distributed throughout the primary GlyRS sequence (Fig. 1B). Nine of 16 mutations segregate with disease in affected families (Fig. 1B), providing strong genetic evidence that these mutations are disease‐causing.

*YARS*, encoding cytoplasmic TyrRS, was the second aaRS gene to be associated with CMT. Two heterozygous missense mutations (G41R and E196K) segregated with disease in two unrelated families affected by dominant intermediate CMT type C (DI‐CMTC). In addition, an in‐frame deletion of 12 nucleotides, resulting in the deletion of four amino acids in TyrRS (153‐156delVKQV), was identified as a de novo mutation in a single patient 34. Recently, an additional missense mutation (D81I) was identified in a single late‐onset CMT patient, putatively intermediate type 35. All four mutations map to the catalytic domain of TyrRS (Fig. 1B).

Next, heterozygous missense mutations in *AARS*, encoding cytoplasmic AlaRS, were reported as causative for CMT type 2N (CMT2N). A R329H mutation segregated with disease in two unrelated families 36. Five additional mutations were subsequently identified in patients with axonal CMT (N71Y and G102R) 37, 38, intermediate CMT (E688G) 39, distal hereditary motor neuropathy (D893N) 40, and a family affected by rippling muscles and cramps that included one member that also exhibited axonal CMT (E778A) 41. These six mutations are distributed throughout the AlaRS primary sequence (Fig. 1B). Importantly, five of the six mutations were shown to segregate with disease.

Finally, mutations in *HARS* and *MARS* were associated with CMT. Four heterozygous *HARS* mutations, all mapping to the catalytic domain of HisRS, segregated with peripheral neuropathy in four unrelated families: T132I, P134H, D175G, and D364Y 42 (Fig. 1B). The associated phenotypic spectrum was broad, encompassing axonal CMT, hereditary motor neuropathy and intermediate CMT. Several additional missense variants in *HARS* have been identified in peripheral neuropathy patients, but their pathogenicity is unclear 43. In a family with late‐onset CMT2, two affected family members were heterozygous for a R618C mutation in *MARS*, but the 85 year‐old mother of the index patient also carried the mutation but was unaffected. Thus, this mutation is either not disease‐causing, or displays incomplete penetrance 44. In addition, a single patient from a late‐onset CMT family and an additional patient from an early‐onset CMT family carried a heterozygous P800T mutation in *MARS* 35, 45. Hence, it is uncertain if these *MARS* variants are pathogenic, as segregation with disease was not reported (Fig. 1B).

Interestingly, whereas heterozygous mutations in cytoplasmic aaRS genes are consistently associated with axonal CMT and its phenotypic variants, homozygous or transheterozygous mutations in these genes typically induce more severe syndromes, often involving multiple organ systems (Table 2). In some cases, peripheral neuropathy can be a component of these severe phenotypes. Lastly, recessive mutations in several mitochondrial aaRS genes give rise to a variety of disorders, which were recently reviewed 13.

**Table 2:** Diseases associated with autosomal recessive mutations in cytoplasmic aaRSs

Gene	Disease	Phenotype	Reference	OMIM #
*GARS*	Systemic mitochondrial disease	Myalgia, cardiomyopathy, persistent elevation of blood lactate and alanine, mild perivascular leukomalacia	99	/
*MARS*	Pulmonary alveolar proteinosis	Severe respiratory distress in early childhood, liver disease	100	615486
*MARS*	Multi‐organ phenotype	Metabolic disorder, hypothyroidism, interstitial lung disease, anemia	101	615486
*HARS*	Usher syndrome type 3B	Progressive vision and hearing loss during early childhood	102	614504
*AARS*	Early infantile epileptic encephalopathy 29	Congenital microcephaly, persistent myelination defect, spasticity, refractory myoclonic epilepsy, loss of peripheral deep tendon reflexes	103	616339
*KARS*	Severe neurological symptoms with intermediate CMT	Intermediate CMT, developmental delay, self‐abusive behavior, dysmorphic features, vestibular Schwannoma	104	613641
				613916
*DARS*	Hypomyelination with brainstem and spinal cord involvement and leg spasticity	Severe spasticity, delayed motor development, nystagmus, mild mental retardation, hypomyelination, and white matter lesions	105	615281
*QARS*	Progressive microcephaly, seizures, and cerebral‐cerebellar atrophy	Progressive microcephaly, severe and intractable seizures in infancy, atrophy of the cerebral cortex and cerebellar vermis and hemispheres	106	615760
*RARS*	Hypomyelinating leukodystrophy 9	Hypomyelination resulting in severe spasticity, nystagmus, mental retardation	107	616140
*LARS*	Infantile liver failure syndrome 1	Acute liver failure, anemia, renal tubulopathy, developmental delay, seizures	108	615438

## Animal models for CMT associated with aaRS mutations

A number of animal models for CMT associated with aaRS mutations (CMT‐aaRS) have been generated, which recapitulate several characteristics of the human disease. Two mouse CMT2D models originated from independent ENU mutagenesis screens 46, 47. In the first model, mice heterozygous for a substitution of Pro278 by Lys and Tyr (P278KY) in GlyRS (corresponding to P234KY in human cytoplasmic GlyRS) exhibited overt neuromuscular dysfunction by three weeks of age and a greatly shortened life span 46. *Gars^P278KY/+^* mice displayed abnormal neuromuscular junction (NMJ) morphology, more pronounced in distal muscles. Neuromuscular transmission was impaired, and nerve conduction velocities reduced. Loss of large diameter peripheral motor and sensory axons was evident, more pronounced distally and without myelination defects or spinal cord pathology 46.

In the second model, mice heterozygous for a GlyRS C201R mutation (corresponding to C157R in human cytoplasmic GlyRS) showed loss of grip strength, diminished fine motor control, and reduced hindlimb muscle force, with an apparently normal life span 47. Muscle atrophy and NMJ morphology defects were evident, and the diameter of peripheral sensory axons was reduced, leading to reduced NCVs 47. Interestingly, NMJ denervation in *Gars^P278KY/+^* and *Gars^C201R/+^* mice is preceded by NMJ maturation defects 48.

Apart from CMT2D mouse models, a number of *Drosophila* CMT‐aaRS models have been reported, which are ideally suited to conduct genetic screens for putative disease‐modifying genes. A *Drosophila* DI‐CMTC model was generated by expression of human TyrRS using the UAS/GAL4 system, which allows for spatial and temporal control of transgene expression 49. In contrast to wild type (WT) TyrRS, expression of CMT‐mutant TyrRS (G41R, 153‐156delVKQV, and E196K) induced progressive motor deficits, electrophysiological evidence of neuronal dysfunction, and axonal degeneration. Not only ubiquitous, but also neuron‐selective expression of mutant TyrRS induced these phenotypes, indicating that the mutant enzymes are intrinsically toxic to neurons 50.

More recently, several independent *Drosophila* CMT2D models have been reported. One set of models involves transgenic overexpression of the cytoplasmic form of *Drosophila* GlyRS with a C‐terminal HA tag, either WT or with G240R or P234KY mutations. Neuron‐selective expression of GlyRS_G240R induced an age‐dependent, mild climbing defect, and selective expression of mutant GlyRS transgenes in the “giant fiber” system 51 triggered electrophysiological and morphological defects in the giant fiber axon terminal 52. In larvae, mutant *Drosophila* GlyRS reduced the frequency of larval body wall muscle contractions when expressed either in muscle, in neurons, or ubiquitously 53. The second set of *Drosophila* CMT2D models utilizes transgenes that allow expression of both the cytoplasmic and mitochondrial forms of human GlyRS, carrying E71G, G240R, G526R, or no mutations 54. Ubiquitous expression of mutant, but not WT, transgenes from the adult stage onwards greatly reduced life span, and motor neuron selective expression impeded climbing behavior and induced NMJ morphology defects and progressive muscle denervation, distal muscles being more severely affected. Selective expression of mutant GlyRS in sensory neurons induced morphology defects 54. Apart from the expression of mixed cytoplasmic and mitochondrial forms of human GlyRS versus cytoplasmic *Drosophila* GlyRS, the two sets of models distinguish themselves by the fact that the human GlyRS transgenes are untagged, and introduced into specific genomic landing sites, which uniformizes transgene expression levels 55, 56. Taken together, the available *Drosophila* and mouse models form complementary tools to study the molecular pathogenesis of CMT‐aaRS and they have significantly contributed to our current understanding of disease pathogenesis.

## How could mutant aaRSs cause peripheral neuropathy?

### Could partial loss of aminoacylation activity underlie CMT‐aaRS?

It was initially hypothesized that CMT‐causing aaRS mutations may lead to loss of aminoacylation activity. Since patients are heterozygous for CMT‐aaRS mutations, this could lead to a reduction of “overall” aminoacylation activity, either through haploinsufficiency or a dominant negative mechanism. This may deplete the pool of aminoacylated cognate tRNAs, so that, when below a critical threshold, the supply of this tRNA species to the ribosome would become insufficient, leading to ribosome stalling at codons for the cognate amino acids, thus inhibiting translation. This is a realistic scenario, as recently shown by a mouse mutant, in which diminished amounts of a brain‐specific Arg‐tRNA^Arg^ causes ribosome stalling at Arg codons, which is exacerbated by the absence of Gtpbp2, a protein functioning to resolve stalled ribosomes, leading to severe neurodegeneration 57. This hypothesis was further supported by the observation that almost all of the amino acid residues mutated in CMT‐aaRS are highly conserved during evolution: 31 of the 34 mutated residues are conserved at least as far as *Drosophila melanogaster* (Table 3). This leads to the stunning observation that in *Drosophila* GlyRS 16 of the 17 CMT‐associated residues are strictly conserved, whereas the overall amino acid identity is only 60%. For TyrRS and HisRS, all disease‐associated residues are at least conserved to yeast (Table 3). This suggests that interference with an ancient, important or even essential function of these enzymes, most probably aminoacylation, may underlie CMT pathogenesis.

**Table 3:** Effect of CMT mutations on aaRS aminoacylation activity

aaRS	Mutation	In vitro aminoacylation assay	Yeast complementation assay	Evolutionary conservation	Reference
GlyRS	A57V	++	ND	Chicken	67
	E71G	+++	+++	Yeast	23, 54, 58, 59
	L129P	−	+	Yeast	23, 58, 59
	D146N	+	++	Yeast	67
	D146Y	ND	ND	Yeast	
	C157R	+++	ND	*C. elegans*	47
	S211F	−	ND	*C. elegans*	67
	L218Q	ND	ND	Yeast	31
	P234KY	+++	+++	Yeast	46, 59
	M238R	ND	ND	Zebrafish	
	G240R	+	+++	*D. melanogaster*	23, 54, 58, 59, 67
	P244L	−	−	Yeast	29
	E279D	ND	ND	Yeast	
	I280F	−	+++	Yeast	67
	H418R	+	−	Yeast	58, 67
	D500N	+++	ND	*D. melanogaster*	59, 67
	G526R	−	−	Yeast	23, 54, 58, 64
	G598A	−	+++	*C. elegans*	65, 67
TyrRS	G41R	−	−	*E. coli*	50, 60
	D81I	ND	ND	Yeast	
	153‐156del VKQV	+	+	Yeast	50, 60
	E196 K	+++	+++	Yeast	50, 60
AlaRS	N71Y	−	−	Yeast	41
	G102R	ND	−	*E. coli*	38
	R329H	−	−	*E. coli*	41
	E688G	ND	ND	*E. coli*	39
	E778A	+++	+++	Rat	41
	D893N	ND	ND	*D. melanogaster*	
HisRS	T132I	ND	−	*E. coli*	42
	P134H	ND	−	Yeast	42
	D175G	ND	+	Yeast	42
	D364Y	ND	−	*E. coli*	42
MetRS	R618C	ND	−	Yeast	44
	P800T	ND	ND	*C. elegans*	

ND, not determined. For GlyRS, the positions of the mutations refer to the cytoplasmic form of the human protein.

It is possible that for some CMT‐aaRS mutations, partial loss of aminoacylation activity may cause or causally contribute to peripheral neuropathy phenotypes. However, at least for some CMT‐aaRS mutations, several lines of evidence have shown that loss of aminoacylation activity is not required to cause CMT. First, direct analysis of aminoacylation activity, either using in vitro aminoacylation assays or in vivo genetic complementation assays in yeast or *Drosophila*, revealed that several CMT‐aaRS mutations result in loss or severe reduction of aminoacylation activity, but some mutations, which segregate with disease in families, do not affect aminoacylation activity, including GlyRS E71G, TyrRS E196K, and AlaRS E778A 41, 50, 54, 58, 59, 60 (Table 3). Furthermore, in CMT2D mouse models, heterozygous P278KY and C201R mutations in GlyRS do not reduce tRNA^Gly^ aminoacylation activity 46, 47, 59. Secondly, if reduction of aminoacylation activity would underlie CMT pathogenesis, transgenic increase of WT aaRS expression should rescue peripheral neuropathy in CMT‐aaRS animal models. This was not the case in CMT2D mouse models 61. Thirdly, in case of a haploinsufficient mechanism, animals heterozygous for aaRS loss‐of‐function alleles should develop peripheral neuropathy. However, heterozygosity for a *Gars* loss‐of‐function allele in mice or a TyrRS null allele in flies did not induce peripheral neuropathy phenotypes 46, 50. Finally, overexpression of mutant human GlyRS in *Drosophila* induced peripheral neuropathy phenotypes, without reduction of tRNA^Gly^ aminoacylation activity and without altering the in vivo ratio of aminoacylated versus non‐aminoacylated tRNA^Gly^ 54. Taken together, this leaves us with two possible scenarios: (i) all CMT‐aaRS mutations result in the acquisition of a novel, toxic property that underlies peripheral neuropathy; or (ii) some CMT‐aaRS mutations cause CMT through a gain‐of‐toxic‐function mechanism, whereas other CMT‐aaRS mutations cause CMT through partial loss of aminoacylation activity, most likely through a dominant‐negative mechanism. Further research is needed to distinguish between these two scenarios.

### tRNA misacylation leading to misincorporation of amino acids in proteins is unlikely to underlie CMT‐aaRS

A second possible mechanism is that CMT‐aaRS mutations could lead to an increased frequency of tRNA misacylation, either by reducing the ability of aaRSs to discriminate cognate from non‐cognate amino acids, or by impairing the pre‐ or post‐transfer editing activity. tRNA misacylation would lead to misincorporation of amino acids in proteins, leading to protein misfolding and aggregation. The plausibility of this mechanism is illustrated by the mouse “sticky” mutant, in which a A734E mutation in the AlaRS editing domain compromises the proofreading activity of this enzyme, resulting in cerebellar Purkinje cell loss and ataxia, and intracellular accumulation of misfolded, ubiquitinated proteins in neurons 62. Similarly, in *Drosophila*, a double mutation in PheRS, which both impairs the capacity to discriminate Phe from Tyr and disrupts the post‐editing activity, leads to misacylation of tRNA^Phe^ with Tyr, resulting in protein mistranslation and ER stress. The mutant flies exhibit several defects, including neuronal loss, impaired locomotor performance, shorter life span, and smaller organ size 63.

However, there are several arguments against this hypothesis. Firstly, some CMT‐aaRS mutations disrupt the binding site for amino acids or ATP, resulting in an aaRS no longer able to activate amino acids, cognate or non‐cognate. This was shown for GlyRS G526R 64 and TyrRS G41R and 153‐156delVKQV mutations 60. With respect to defective editing, of the five CMT‐associated aaRSs, only AlaRS has post‐editing activity, while MetRS and AlaRS have pre‐editing activity (Table 1). CMT mutations in these editing aaRSs have not been reported to affect the editing process. In particular, the AlaRS_E778A mutation affects neither aminoacylation nor editing activity 41. Finally, sticky mice exhibit cerebellar ataxia but no peripheral neuropathy. Vice versa, *Gars^P278KY/+^* mice do not show cerebellar neurodegeneration, and there is no evidence for accumulation of misfolded proteins in these mice 65. Thus, it seems unlikely that translational infidelity due to tRNA misacylation underlies CMT‐aaRS.

### Alteration of aaRS dimerization is unlikely to cause CMT‐aaRS

Interestingly, when mapped on the GlyRS crystal structure, CMT2D‐causing mutations cluster around the dimer interface, suggesting that alteration of GlyRS dimer formation may be involved in disease pathogenesis 59. However, different CMT‐GlyRS mutations have different effects on dimerization: some mutations strengthen dimer formation, others prevent dimer formation, and yet others do not influence dimerization 59, 64, 66. Therefore, it is unlikely that altered dimer formation causes CMT2D.

### Could mislocalization of mutant aaRSs contribute to CMT‐aaRS?

The fact that loss of aminoacylation activity is not required to cause CMT does not exclude the possibility that CMT‐associated mutations could result in subcellular mislocalization of aaRSs, possibly causing defects in local protein translation. For instance, reduced localization of CMT‐mutant aaRSs to axons and/or nerve endings could lead to impaired local protein synthesis and axonal degeneration. This is a conceivable scenario, as endogenous GlyRS and TyrRS proteins are localized to motor neuron cell bodies, axons and nerve endings in human and mouse nervous tissues, and in cultured mouse motor neurons 34, 58, 65. Consistently, a number of CMT‐mutant GlyRS and TyrRS proteins displayed altered subcellular distribution in neuronal cell lines, which can be differentiated to form neurite projections. Specifically, in the mouse motor neuron, neuroblastoma fusion cell line MN‐1, some CMT GlyRS mutants exhibited altered subcellular localization, including L129P, S211F, P234KY, G240R, P244L, I280F, H418R, and G598A GlyRS 58, 65, 67. However, other GlyRS mutants (A57V, E71G, D146N, D500N, and G526R) showed a similar subcellular distribution as WT GlyRS 58, 67.

In differentiating N2a mouse neuroblastoma cells, CMT‐mutant TyrRS (G41R and E196K) or GlyRS (L129P, P234KY, G240R, H418R, D500N, and G526R) displayed a reduced distribution to neurite tips 34, 59. In contrast, WT and E778A AlaRS displayed a similar subcellular localization pattern in MN‐1 cells 41.

Different from studies in cell lines, the subcellular localization of mutant GlyRS and TyrRS in CMT animal models was generally reported to be indistinguishable from the respective WT proteins. In CMT2D mouse models, GlyRS localization in sciatic nerve fibers and in spinal cord sections was unaltered 65. However, in vivo expression of WT, L129P, or G240R human GlyRS in mouse motor and sensory neurons by viral gene transfer revealed WT GlyRS localization along the length of sciatic nerve axons, whereas localization of L129P and G240R GlyRS to sciatic nerve axons was diminished 68, 69. In *Drosophila* CMT2D models, HA‐tagged WT and P234KY *Drosophila* GlyRS showed similar subcellular localization in giant fiber axons and motor neuron cell bodies and axons 52, 53, and WT, E71G, G240R, and G526R human GlyRS displayed similar subcellular distribution in motor neurons, with diffuse localization to motor neuron cell bodies, axons, and NMJs 54. Furthermore, in *Drosophila* DI‐CMTC models, WT, G41R, 153‐156delVKQV, and E196K human TyrRS displayed similar distribution in both motor and sensory neurons, with homogeneous distribution throughout the cell body, axon, and major dendrite branches 54.

The discrepancy between in vitro and in vivo studies may partly be due to effects of protein tagging and overexpression, as well as the fact that aaRS distribution may differ between differentiating neuronal cells in vitro and mature motor and sensory neurons in vivo. For instance, the GlyRS P234KY mutant mislocalizes in MN‐1 and N2a cells, but displays normal subcellular localization in the mouse spinal cord 59, 65. Importantly, even in neuronal cell lines, some CMT‐mutant aaRSs display normal subcellular localization, including GlyRS A57V, E71G, D146N, and AlaRS E778A. Thus, at least for these mutants, defects in local protein translation due to subcellular mislocalization are unlikely. Furthermore, the peripheral neuropathy phenotypes in mouse and *Drosophila* CMT‐aaRS models are not attributable to subcellular mislocalization of mutant aaRSs. Further studies are needed to evaluate whether for some mutant aaRSs, subcellular mislocalization could contribute to CMT pathogenesis. In this respect, it would be highly interesting to investigate the subcellular localization of mutant aaRSs in CMT‐aaRS patient tissues.

### Could interference with non‐canonical functions of aaRSs cause peripheral neuropathy?

As indicated above, several aaRS have acquired additional functions during evolution, often through incorporation of additional functional domains. For the CMT‐associated aaRSs, functions beyond aminoacylation have been described for GlyRS, TyrRS, MetRS, and HisRS 70, 71, 72, 73, 74, 75, 76, 77, and CMT‐causing mutations could possibly interfere with these functions. The fact that missense mutations in aaRSs can affect non‐canonical functions is illustrated by a Y341A mutation in TyrRS, which uncovers an internal ELR tripeptide, thereby activating the cytokine function in the full‐length TyrRS protein, which is normally inactive as a cytokine 78. Unfortunately, the effect of CMT‐causing mutations on GlyRS, TyrRS, MetRS, and HisRS non‐canonical functions has thus far not been investigated. Although this mechanism might contribute to CMT pathogenesis for some mutations, it seems unlikely that it represents a common pathogenic mechanism underlying CMT‐aaRS, for a number of reasons. First, for AlaRS, non‐canonical functions have not been reported. Secondly, different aaRSs acquired distinct non‐canonical functions. Thus, if alteration of non‐canonical functions would underlie CMT‐aaRS, this would imply that different mutant aaRS would cause CMT through distinct molecular mechanisms. Although not impossible, this seems unlikely. Furthermore, for GlyRS, CMT mutations are distributed throughout the protein, making it unlikely that all mutations affect GlyRS non‐canonical functions. Thus, to date, no concrete data suggest that alteration of non‐canonical aaRS functions contribute to CMT pathogenesis.

### A gain‐of‐toxic‐function mechanism likely underlies CMT‐aaRS

In CMT2D mouse models, convincing genetic evidence indicates that mutant GlyRS proteins cause peripheral neuropathy by a “toxic‐gain‐of‐function” mechanism. Genetically, the characteristics of a toxic‐gain‐of‐function (neomorphic) allele are that phenotypes are not modified by altering the levels of WT protein, but enhanced by increasing the levels of mutant protein. Consistently, transgenic overexpression of WT GlyRS does not improve the neuropathy phenotype in heterozygous *Gars^P278KY/+^* and *Gars^C201R/+^* mice 61. Furthermore, homozygous *Gars^C201R/C201R^* and transheterozygous *Gars^C201R/P278KY^* mice in a WT GlyRS overexpression background display enhanced peripheral neuropathy phenotypes 61. Similarly, in *Drosophila* DI‐CMTC and CMT2D models, the severity of peripheral neuropathy phenotypes is transgene dosage‐dependent 50, 53, 54. Molecularly, a toxic‐gain‐of‐function mechanism can involve novel protein‐protein interactions enabled by the disease‐causing mutations, in which the WT protein does not engage. These novel protein‐protein interactions could affect the function of the interacting protein(s), thereby causing disease. Interestingly, several spatially dispersed GlyRS mutations (L129P, G240R, G526R, and G598A) induce the same conformational opening of a consensus area that is mostly buried in WT GlyRS 66. A possible molecular mechanism underlying the toxic‐gain‐of‐function of CMT‐mutant GlyRS was recently reported 79, as several CMT‐GlyRS mutants, including E71G, L129P, P234KY, and G240R, strongly bound to neuropilin‐1 (Nrp1), a co‐receptor for both semaphorins and VEGF‐A 80. In contrast, WT GlyRS only weakly bound to Nrp1. VEGF‐A was previously implicated in motor neuron degeneration, as low VEGF‐A expression leads to adult‐onset motor neuron degeneration in mice, reminiscent of human amyotrophic lateral sclerosis (ALS) 81, and exogenous VEGF‐A administration has significant therapeutic effects in ALS rodent models 82, 83. CMT‐GlyRS mutants competed with VEGF‐A for binding to Nrp1, and heterozygosity for Nrp1 enhanced the peripheral neuropathy phenotype of *Gars^P278KY/+^* mice. Furthermore, increasing VEGF‐A expression in hindlimb muscles improved motor performance of *Gars^P278KY/+^* mice 79. Although it remains to be investigated whether all CMT‐causing mutations increase GlyRS binding to Nrp1, this mechanism illustrates how CMT‐mutant, misfolded GlyRS may interfere with signaling pathways that are critical for survival of peripheral motor and sensory axons. It is likely that other CMT‐mutant aaRSs harbor similar neomorphic activities, and unraveling their molecular mechanisms is a major challenge that the CMT‐aaRS field is currently facing. Of note, the currently available evidence does not exclude the possibility that some CMT‐aaRS mutant proteins might cause peripheral neuropathy through partial loss of aminoacylation activity.

### Inhibition of protein translation independent of aminoacylation: A common pathogenic mechanism of CMT‐aaRS?

**Figure 2:**
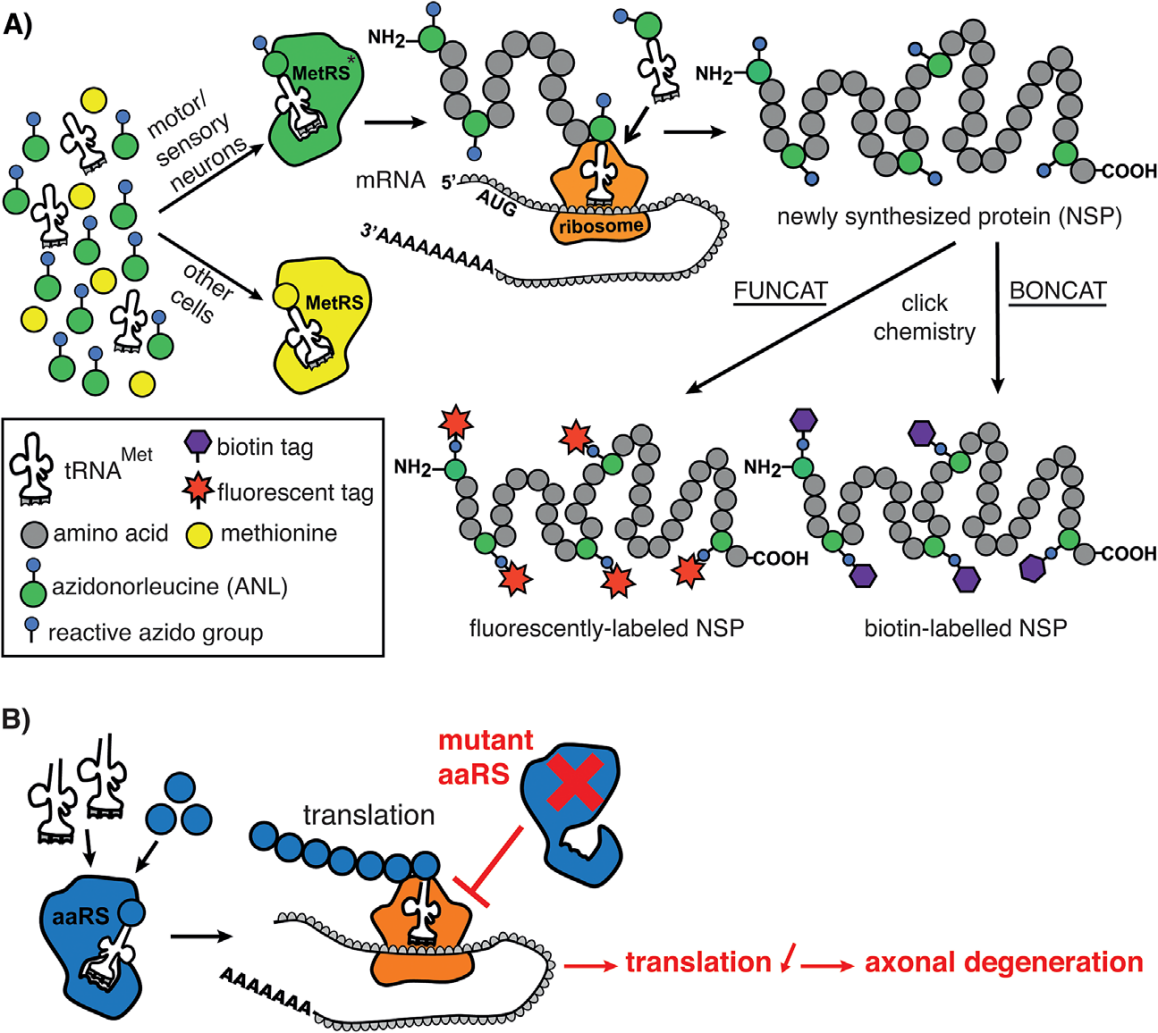
Impaired protein translation in *Drosophila* CMT‐aaRS models. **A:** Non‐canonical amino acid tagging (NCAT) for cell‐type‐specific labeling of proteomes in *Drosophila*. In contrast to endogenous MetRS, a modified MetRS (MetRS*) is able to aminoacylate tRNA^Met^ with the non‐canonical amino acid azidonorleucine (ANL). When transgenic *Drosophila* that cell‐type specifically express MetRS* are fed with ANL, ANL will be incorporated in newly synthesized proteins (NSPs) in cells that express MetRS*. After a defined labeling time, relevant tissues are dissected and ANL‐containing proteins are labeled by “click chemistry” with either a fluorescent (FUNCAT) or a biotin tag (BONCAT). Quantification of tagged proteins by fluorescence microscopy or western blot allows to determine the relative amounts of NSPs, which are proportional to the protein synthesis rate. **B:** CMT‐mutant tRNA synthetases inhibit translation independent of aminoacylation, leading to degeneration of peripheral motor and sensory axons.

The toxic‐gain‐of‐function mechanism may involve aberrant interactions with components of the protein translation pathway or, alternatively, with pathways unrelated to translation. The hypothesis that mutant aaRSs could affect translation was recently investigated in *Drosophila* CMT2D and DI‐CMTC models, utilizing a novel method based on non‐canonical amino acid tagging, which allows to cell‐type‐specifically monitor translation in vivo (Fig. 2A) 54, 84. Remarkably, selective expression of three distinct GlyRS mutants (E71G, G240R, G526R) in either motor or sensory neurons dramatically inhibited global protein translation 54. ^35^S‐methionine incorporation confirmed impaired translation when mutant GlyRS was ubiquitously expressed in adult flies. Furthermore, selective expression of three distinct CMT‐TyrRS mutants (G41R, del153‐156VKQV, E196K) also significantly inhibited translation in motor and sensory neurons, suggesting that impaired translation may constitute a common pathogenic mechanism underlying CMT‐aaRS 54.

Consistent with a toxic‐gain‐of‐function mechanism, inhibition of translation by mutant GlyRS was independent of tRNA^Gly^ aminoacylation. Indeed, expression of mutant GlyRS transgenes – in an otherwise WT *Drosophila* *GlyRS* background – did not reduce overall aminoacylation activity and did not alter the in vivo ratio of glycylated versus non‐glycylated tRNA^Gly^, with >90% Gly‐tRNA^Gly^. Moreover, co‐overexpression of WT *Drosophila* GlyRS did not rescue the translation defect induced by mutant human GlyRS 54. Finally, it is likely that impaired translation causally contributes to peripheral neuropathy phenotypes in *Drosophila* CMT‐aaRS models, as inhibition of translation independent of mutant aaRS expression – by expression of constitutively active forms of eukaryotic initiation factor 4E binding protein (4E‐BP) – was sufficient to induce muscle denervation and sensory neuron morphology defects 54. Thus, mutant aaRSs inhibit translation by a toxic‐gain‐of‐function mechanism, independent of aminoacylation, and impaired translation may causally contribute to peripheral neuropathy phenotypes (Fig. 2B). It will now be important to evaluate whether translation is also affected in mouse CMT2D models, and to identify the molecular mechanism by which mutant aaRSs inhibit translation (Box 2).

Box 2 **Proposed experimental approaches to verify the hypothesis**

The central hypothesis proposed here is that inhibition of protein translation is a common pathogenic mechanism underlying CMT‐aaRS. In order to test this hypothesis, several aspects would need experimental verification:

1. To determine whether all CMT‐aaRS mutant proteins inhibit translation in vivo, it would be necessary to generate additional CMT‐aaRS animal models, including CMT‐AlaRS and CMT‐HisRS models. NCAT technology could then be used to evaluate whether protein translation is inhibited in motor and sensory neurons of these models.
2. To study the relevance of the findings in *Drosophila* models for human CMT‐aaRS, it should be evaluated whether protein translation is also affected in CMT‐aaRS mouse models, and/or in induced pluripotent stem cell (iPSC)‐derived motor and sensory neurons from CMT‐aaRS patients.
3. A first step to gain insight into the molecular mechanism underlying the translation defect in CMT‐aaRS *Drosophila* models could be to determine whether CMT‐mutant aaRSs interfere with upstream regulatory pathways of translation, or rather directly with translation initiation or elongation. This could be done by genetic manipulation of known key regulators of these processes in *Drosophila* CMT‐aaRS models, and evaluating the effect on protein translation by NCAT.

More broadly, it will be important to determine whether all CMT‐mutant aaRSs cause disease through a gain‐of‐toxic‐function mechanism, or, alternatively, whether some mutant aaRSs cause disease through loss‐of‐function and some through gain‐of‐toxic‐function. This could be done by generating additional CMT‐aaRS animal models and testing whether overexpression of the relevant wild type aaRS rescues the peripheral neuropathy phenotypes.

Interestingly, regulation of translation by aaRSs through non‐catalytic mechanisms have been previously reported. Some aaRSs regulate the translation of their own transcript, others regulate translation of a select number of transcripts different from their own, and phosphorylation of MetRS provides a mechanism for global regulation of translation 85. For instance, *E. coli* ThrRS binds as a homodimer to two stem‐loop structures in the 5′UTR of its own mRNA, which mimic the anticodon arm of tRNA^Thr^ 86, 87, thereby preventing ribosome binding and inhibiting translation initiation 88. As tRNA^Thr^ and ThrRS mRNA compete for binding to ThrRS, this constitutes a negative autoregulatory mechanism 88, 89, 90. Negative autoregulation of translation was also reported for yeast AspRS, which binds to the 5′UTR of its transcript that adopts a tRNA^Asp^ anticodon‐like structure 91, 92.

Gene‐specific regulation of translation by aaRSs is exemplified by GluProRS, which is a component of the interferon‐gamma‐activated inhibitor of translation (GAIT) complex. This heterotetrameric complex suppresses translation of selected mRNAs in interferon‐gamma‐activated monocytic cells 93. In response to interferon‐gamma, GluProRS is phosphorylated and released from the MSC and incorporated in the GAIT complex, in which it is the subunit that binds to the 3′UTR of target mRNAs, resulting in translational silencing of target mRNAs 94. In this case, tRNA mimicry is not involved in target mRNA binding, as the WHEP domains in GluProRS are responsible for binding of the GAIT element stem loop 95. Interestingly, an additional level of translational regulation of GAIT target genes involves the production of a truncated form of GluProRS, which shields GAIT‐element bearing transcripts from the GAIT complex, thereby countering translational repression 96. The fact that aaRSs can not only inhibit, but also activate translation is illustrated by the binding of GlyRS to the poliovirus IRES, which promotes the accommodation of the ribosome and greatly enhances IRES activity. Poliovirus IRES uses tRNA^Gly^ anticodon stem‐loop mimicry to recruit GlyRS 97.

Finally, phosphorylation of human MetRS on Ser^662^ by GCN2 reduces its catalytic activity due to diminished tRNA_i_^Met^ binding, leading to downregulation of global translation 73. Since expression of CMT‐mutant GlyRS and TyrRS in *Drosophila* motor and sensory neurons inhibited global rather than gene‐specific translation, only the latter mechanism may possibly mediate translational inhibition in *Drosophila* CMT models. However, since the in vivo ratio of aminoacylated versus non‐aminoacylated tRNA_i_^Met^ was unaltered in larvae that ubiquitously expressed mutant GlyRS 54, it seems unlikely that this mechanism is involved.

## Conclusions and prospects

Over the past 12 years, dominant mutations in five distinct aaRS genes have been associated with CMT peripheral neuropathy, and significant progress has been made toward understanding how mutations in these ubiquitously expressed, essential enzymes may lead to selective degeneration of peripheral motor and sensory axons. It became evident that some CMT‐causing aaRS mutations do not affect aminoacylation activity, showing that loss of aminoacylation activity is not required to cause peripheral neuropathy. Furthermore, mislocalization of CMT‐mutant aaRSs has been reported in cultured neuronal cell lines, but not in CMT‐aaRS animal models, and is, therefore, not necessary to induce CMT phenotypes. It is further unlikely that misincorporation of amino acids in proteins due to tRNA misacylation contributes to CMT‐aaRS pathogenesis. Rather, convincing genetic evidence in CMT‐aaRS animal models has shown that a gain‐of‐toxic‐function mechanism underlies CMT‐aaRS pathogenesis, and interference with VEGF‐Nrp1 signaling is a possible molecular mechanism contributing to CMT‐GlyRS. Moreover, impaired translation may be a common pathogenic event in CMT‐aaRS, as all of six CMT‐mutant GlyRS and TyrRS proteins inhibited translation in *Drosophila* motor and sensory neurons. This translational slowdown was independent of tRNA aminoacylation and caused by a gain‐of‐toxic‐function mechanism. The molecular mechanism by which mutant aaRSs inhibit translation should be the focus of future research (Box 2). It is further important to confirm that translational defects are also present in CMT‐aaRS mouse models (Box 2). Finally, another outstanding question is to which extent distinct mutations in distinct aaRSs cause peripheral neuropathy through common molecular mechanisms (Box 2). For instance, it remains possible that for some mutations, loss of aminoacylation activity may contribute to axonal degeneration. Overall, if future research could provide detailed molecular insights into CMT‐aaRS pathogenesis, this may form the first step toward the development of an effective drug treatment for this incurable disorder.

The author has declared no conflict of interest.
